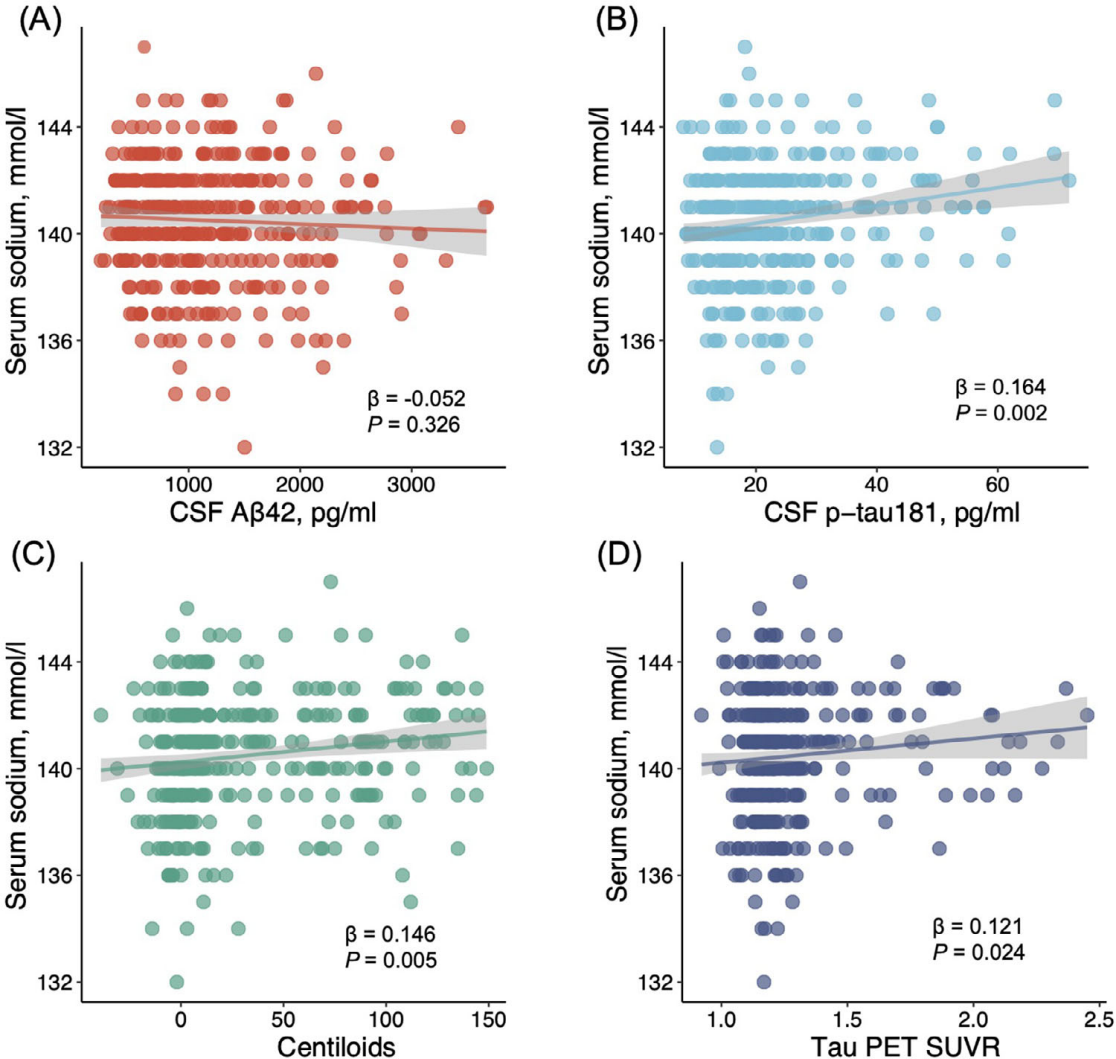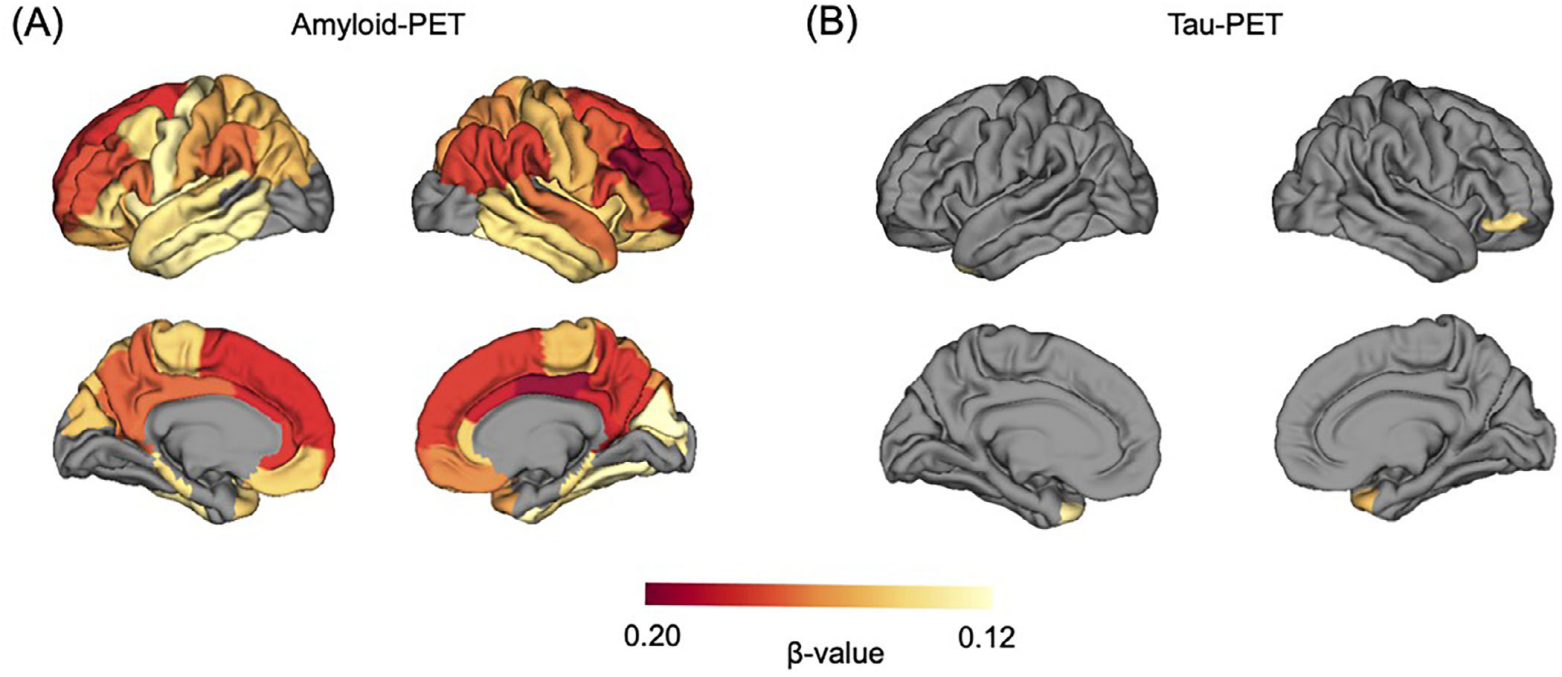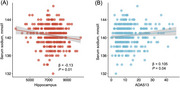# Serum sodium is associated with amyloid‐dependent tau pathology, neurodegeneration, and cognitive impairment in Alzheimer's disease

**DOI:** 10.1002/alz70856_101607

**Published:** 2025-12-25

**Authors:** Yuhan Chen, Zhiqi Mao

**Affiliations:** ^1^ Hebei North University, Zhangjiakou, Hebei, China; ^2^ Chinese PLA General Hospital, Beijing, Beijing, China

## Abstract

**Background:**

Vascular dysfunction plays a role in Alzheimer's disease (AD) pathophysiology. While sodium is vital for vascular function, its involvement in AD pathology needs clarification.

**Method:**

We analyzed 353 participants from the Alzheimer's Disease Neuroimaging Initiative (ADNI), examining serum sodium levels, cerebrospinal fluid (CSF) and positron emission tomography (PET) biomarkers, magnetic resonance imaging (MRI), and cognitive function. An additional independent sample (*N* = 471) with CSF sodium‐related proteins and AD biomarkers was studied. Linear regression models evaluated associations between serum sodium and AD pathology, neurodegeneration, and cognition. Spearman's correlation analyzed relationships between CSF sodium‐related proteins and AD biomarkers.

**Result:**

Higher serum sodium levels correlated with increased AD pathology, reduced hippocampal volume, and greater cognitive decline (all *p* < 0.05). Amyloid PET showed this relationship in AD‐susceptible regions, including neocortex and limbic system. High serum sodium groups showed higher tau pathology, lower hippocampal volume, and worse cognitive decline per unit increase in amyloid PET versus low serum sodium groups (all *p* < 0.05). Among 14 CSF sodium‐related proteins, six correlated with CSF AD pathology and amyloid PET, while two correlated with hippocampal volume and cognitive function. Sodium channel subunit beta‐2 (SCN2B) and beta‐3 (SCN3B) showed strongest correlations.

**Conclusion:**

Serum sodium plays a crucial role in AD progression, revealing a potential network of sodium dysregulation in AD pathology. Targeting sodium may provide a novel therapeutic approach for slowing AD progression, particularly in impeding amyloid‐related downstream events.